# Omics in the Red Palm Weevil *Rhynchophorus ferrugineus* (Olivier) (Coleoptera: Curculionidae): A Bridge to the Pest

**DOI:** 10.3390/insects14030255

**Published:** 2023-03-04

**Authors:** Manee M. Manee, Fahad H. Alqahtani, Badr M. Al-Shomrani, Hamadttu A. F. El-Shafie, Guilherme B. Dias

**Affiliations:** 1 National Center for Bioinformatics, King Abdulaziz City for Science and Technology, Riyadh 11442, Saudi Arabia; 2 Institute of Advanced Agricultural and Food Technologies, King Abdulaziz City for Science and Technology, Riyadh 11442, Saudi Arabia; 3 Date Palm Research Center of Excellence, King Faisal University, Al-Ahsa 31982, Saudi Arabia; 4 Brazilian Ministry of Health, Brasília 70723040, Brazil

**Keywords:** red palm weevil, insect pests, draft genome, transcriptomics, metagenomics

## Abstract

**Simple Summary:**

The red palm weevil (RPW) *Rhynchophorus ferrugineus* (Coleoptera: Curculionidae) is a species of beetle that depends on palm trees to complete its life cycle. RPW larvae feed on the palm tree trunk and can result in tree death, thus having a major impact on both wild and cultivated palm trees in several countries. The resulting economic and biodiversity losses caused by invasive populations of the RPW gave it a major pest status, and now governments, companies, and researchers across multiple countries are interested in developing effective control strategies to prevent further damage. Managing agricultural pests in the face of pressures such as climate change, rising levels of insecticide resistance, and increasing demands for food production will require advanced knowledge of pest biology. In this review, after presenting basic aspects of RPW biology and pest status, we gather information on the use of omics technologies, such as transcriptomics, genomics, and metagenomics, and explore their achievements and potentials to illuminate RPW biology and improve management. We finalize the review by exploring current opportunities and challenges in the use of these technologies for RPW management.

**Abstract:**

The red palm weevil (RPW), *Rhynchophorus ferrugineus* (Coleoptera: Curculionidae), is the most devastating pest of palm trees worldwide. Mitigation of the economic and biodiversity impact it causes is an international priority that could be greatly aided by a better understanding of its biology and genetics. Despite its relevance, the biology of the RPW remains poorly understood, and research on management strategies often focuses on outdated empirical methods that produce sub-optimal results. With the development of omics approaches in genetic research, new avenues for pest control are becoming increasingly feasible. For example, genetic engineering approaches become available once a species’s target genes are well characterized in terms of their sequence, but also population variability, epistatic interactions, and more. In the last few years alone, there have been major advances in omics studies of the RPW. Multiple draft genomes are currently available, along with short and long-read transcriptomes, and metagenomes, which have facilitated the identification of genes of interest to the RPW scientific community. This review describes omics approaches previously applied to RPW research, highlights findings that could be impactful for pest management, and emphasizes future opportunities and challenges in this area of research.

## 1. Introduction

The date palm (*Phoenix dactylifera* L.) is one of the oldest cultivated fruit trees in the world and the most extensively cultivated plant in arid and semi-arid regions [1]. It is of major socioeconomic importance because of its nutritional, health, environmental, and social value. Many insect pests attack date palms, causing severe infections and adversely affecting the quality and yield of dates. Among these, the red palm weevil (RPW) is the most important, relying on date palms as its host species and inflicting the most economic damage.

The RPW, whose scientific name is *Rhynchophorus ferrugineus* (Olivier, 1790) (Coleoptera: Curculionidae), is a pest species indigenous to countries in the south and southeast regions of Asia. It was first noticed in the Gulf region in the late 20th century, where it caused severe damage to palm trees; later, it started expanding its range, significantly affecting other regions [2]. In the past few decades, it has spread to multiple regions, affecting countries in North Africa, the Middle East, some parts of the Caribbean, Central America, and the Mediterranean [3]. Due to causing significant destruction of palm plantations around the world, the RPW has become a major agricultural problem [4]. The cost of RPW control in palm farms approaches 8 million USD annually in Saudi Arabia alone [5], underscoring the need to identify sustainable means of dealing with its spread.

Conventional pest management strategies such as chemical pesticides have been utilized for decades to control insect pests that attack food crops; however, the substances in a pesticide may pose hazards to human health and have off-target effects on other species, including natural enemies of the pest, harming the environment, and sometimes resulting in even more infestations following their use [6,7]. Due to the risks associated with using chemical pesticides, governments have been encouraged to move toward safer and more sustainable approaches of pest management. These new approaches are often tailored to the target pest species and may require a deeper knowledge of its biology to be developed. In this context, omics tools could play an essential role in helping protect agricultural crops and native species from insect pests. Through the use of new technologies such as genomics, transcriptomics, and metagenomics, insights can be gained into the molecular mechanisms of insect adaptation to new climates, resistance to pesticides, host switching, population dynamics, and more. Thus, these technologies can reveal mechanisms that may help control pests, manage their resistance development, and recover the environment.

This paper aims to review the currently available literature on RPW omics as a means of identifying gaps in the knowledge and providing perspective on new areas for development. We start by reviewing RPW’s basic biology, pest status, and current management strategies. Then, we review transcriptome-based gene discovery efforts, whole-genome sequencing, metagenomics, and repeat analysis. Finally, we discuss the practical applications of omics-based approaches to RPW’s pest management.

## 2. *R. ferrugineus* Is the Most Damaging Insect Pest of Palms in the World

### 2.1. Life Cycle and Biology

**Figure 1:**
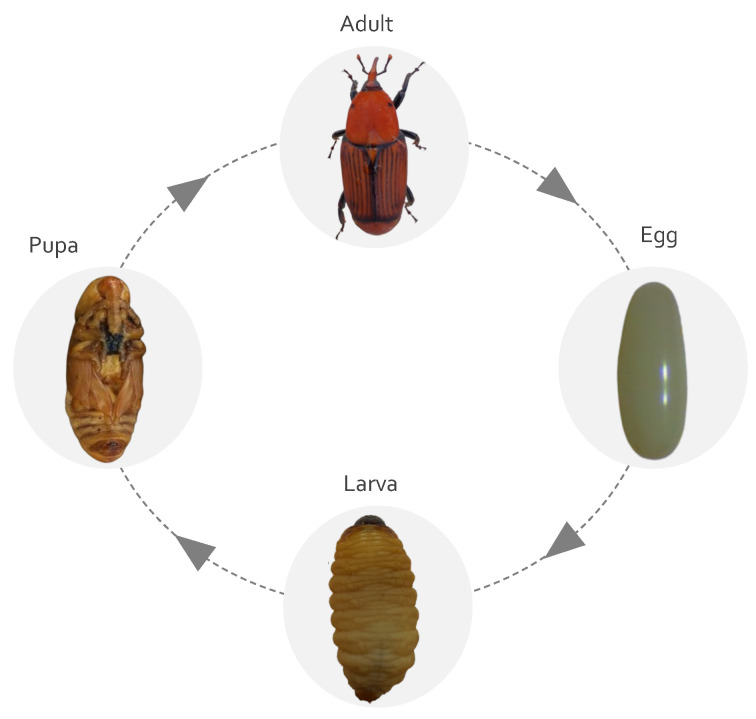
The life cycle stages of the red palm weevil, *R. ferrugineus*.

RPW is a holometabolous insect, and the life cycle passes through four distinct stages; namely egg, larva, pupa, and adult (Figure 1). The female has a high fecundity, and it may deposit as many as 200–250 eggs during its lifespan. Depending on the type of host and environmental conditions, the RPW could produce eggs four times annually. The details of the weevil morphology and life cycle have been studied by many authors [8,9,10,11,12,13].

The pupal stage is usually categorized into two periods. The first is the pre-pupal stage, which lasts for three days, during which the pupa is approximately 25 to 35 mm in size. In the second pupal period, which takes from 12 to 20 days, the pupa initially transforms from a cream to brownish color [9]. As its development progresses, it forms a shiny surface that is greatly furrowed and reticulated. The average size of the pupa at this stage is about 35 × 15 mm. In the end, the RPW emerges as a red-brown adult with a long curved rostrum [10]. The male RPW has a patch of short brown setae in its rostrum, while the female has a bare rostrum that is more slender and slightly longer. The adult stays in its cocoon for an average of eight days and becomes sexually mature during this period of inactivity. The adult female lives for 76 days, while the male lives for 113 days [11]. Adults are great fliers and can travel long distances [12], and during their lifespan, a female RPW can give rise to more than five million weevils in four generations [13].

### 2.2. Pest Status

The RPW is a major insect pest that endangers native palm trees in multiple countries, as well as the sustainability of the crop and ornamental palm industries. It is one of the major invasive pest species globally, attacking over 30 species of palm trees. More precisely, the host range of the RPW expanded from only four species during the mid-1950s [14] to 40 species in several palm-tree-growing regions of the world [15]. Being a polyphagous cryptic internal tissue feeder has given the RPW the advantage of movement and expansion into new geographical areas through trading of infested palms, allowing these invasive populations to access additional palm species [13]. Furthermore, the adaptation of RPW to low temperatures [16] has also aided in the colonization of new habitats.

**Figure 2:**
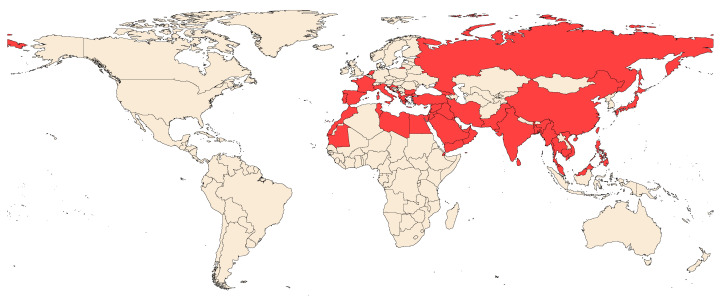
Geographic distribution of the red palm weevil, based on data from the Invasive Species Compendium. Available at https://www.cabi.org/isc/datasheet/47472 (Accessed 31 January 2023).

*R. ferrugineus* affects key agricultural and ornamental palms, such as date palms, coconut palms, Washingtonia palms, oil palms, and Canary Island palms. Its native range is thought to comprise Southeast Asia and Melanesia [2]; it began affecting the Gulf region in the late 1900s, later spreading to many other parts of the world [15]. The RPW has a great capacity for flying, reaching up to 50 km per day [17]; in addition, the ‘concealed boring’ part of its life cycle greatly contributes to its invasive potential, with the RPW mainly spreading through the movement of infested plant materials [18]. The weak quarantine procedures for ornamental plant material movement across countries, as well as slowness in classifying the RPW as a global threat, have facilitated its rapid spread [19]. Due to the movement of infested palms and palm offshoots for the production of dates or landscaping, the weevil has, over the last 30 years, invaded 49 countries in Asia, Europe, Africa, and the Caribbean [3] (Figure 2).

**Figure 3:**
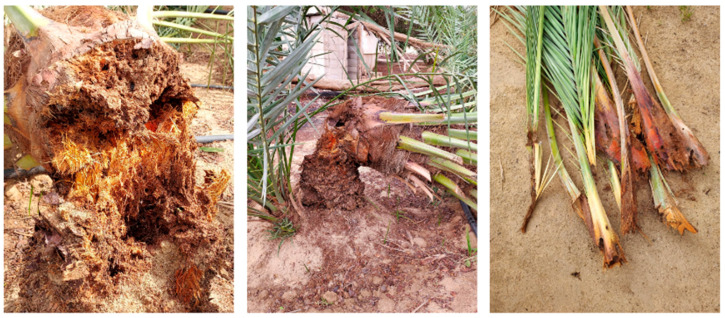
A severely infested date palm.

Although new technologies are being developed for early infestation detection [20], it is often the case that by the time visible signs of infestation appear, damage to the plant is already severe, rendering any attempts to save the tree futile (Figure 3). Damage symptoms on date palm (*Phoenix dactylifera*) have been described in detail [21,22]; the main symptoms include the oozing of brownish viscous fluid together with frass (palm tissue excreted by feeding larvae) that has a typical fermented odor, drying of outer leaves and fruit bunches, drying of infested offshoots, topping of the trunk in the case of severe and extensive tissue damage, and the presence of adults and pupae at the base of the fronds and on the ground nearby [13,23].

The date palm is a key fruit crop in arid regions, and the destruction of palm trees significantly impacts the region’s agriculture and the livelihoods of farmers. For instance, over 80,000 date palm trees in Saudi Arabia are infested with RPW, amounting to an economic burden of over 8 million USD spent on management and eradication with 5% infestation [5,24,25]. Most affected countries have relied on synthetic pesticides and field traps to control the spread of the RPW [26]. However, indiscriminate use of insecticides might not be a sustainable strategy as they can be a danger to biological diversity. Additionally, insecticide resistance has been reported for decades, including in RPW populations [27,28]. In lieu of conventional methods, a series of ideas have been implemented to help eradicate the RPW and enable the survival of palm trees [29]. The Canary Islands of Spain have successfully eradicated red palm weevils using a series of scientifically-based programs supported by adequate resources [30].

### 2.3. Current Management Measures

Due to the high value of date palms as a cash crop and the seriousness of RPW as a pest, the tolerance threshold is set at a low pest population, and 1% infestation is enough to warrant the initiation of control measures [13]. Early detection of infestation represents the most essential step in achieving effective control of the RPW. However, no effective devices or technologies are currently available for field application, and detection of the weevils depends mainly on the experience of qualified personnel, making the whole process laborious, expensive, time-consuming, and inefficient in most cases.

A sequential sampling plan was developed to accurately assess the status of the pest in coconut and date palms based on infestation reports, with the action threshold, RPW aggregation index, and risk of making the wrong decision all built into the plan [31,32]. The weevil is currently managed through mass trapping of adults, routine inspection of farms for early detection, removal of highly-infested palms, chemical treatments, and quarantine measures to prevent the entry of the weevil into new, uninfested areas [3]. Mass trapping can remove large numbers of adult weevils, but the efficiency is relatively low [33], necessitating simultaneous deployment of other methods. The combination of trapping and spatial analysis with Geographical Information System (GIS)-based methods can be used to determine infestation levels, predict the pest’s projected spread, and provide input for risk assessment models; in this capacity, GIS has proven to be useful in monitoring populations, assessing infestations, and predicting, managing, and fighting the spread of pests and diseases. Thus, it can enhance and support decision-making capabilities in RPW management [34].

Pheromone-baited traps are widely used in RPW management programs; however, over-reliance on pheromone trapping and neglect of other strategies leads to a build-up of weevil populations and eventual outbreaks [35]. The four-window black-colored bucket trap is popular in the Middle East and Gulf countries, while the dome-shaped conical Picusan^TM^ trap is commonly used in several European countries [3]. When using food-baited RPW pheromone traps, it is of utmost importance to adopt the best trapping protocols with respect to trap design, color, density, servicing (periodic renewal of food bait), placement, lure attraction, and longevity.

Alongside trapping, preventive and curative chemical treatments are essential for the efficient management of RPW [21,36]. Curative insecticidal treatment of RPW-infested palms in the early stage of infestation represents an integral part of the management strategy; caught early, infested palms often recover after treatment [37].

Recently, microwave radio frequencies were also investigated as a potential method for controlling weevil infestations. This method was shown to result in high weevil mortality, as well as lower fertility of the surviving weevils [38,39].

## 3. Omics Technologies and Their Impact on Pest Science

Pest control will remain crucial in securing a food supply that meets ever-increasing global demands [40]. Yet, even the most sophisticated control programs can be rendered ineffective by pest adaptations. Phenomena such as insecticide resistance and adaptation to new climates occur frequently; however, the processes by which most agricultural pests quickly adapt to or completely evade control strategies are still poorly understood [41]. Because of this general lack of biological knowledge, management programs struggle to develop adequate tools for the challenges they face. In this context, new high throughput ‘omics’ technologies provide an attractive avenue for the advancement of pest biological research. These technologies are characterized by the production of high-resolution and high-volume biological data at relatively low costs.

### 3.1. Omics in Beetles

The advancement of omics tools, such as genomics and transcriptomics, has permitted the emergence of new agricultural pest model organisms. Until recently, the red flour beetle *Tribolium castaneum* was the only taxa from the species-rich order Coleoptera to be well-studied at the genomic level. Since its publication in 2008, the paper describing the *T. castaneum* genome has been cited over 1000 times, reflecting the usefulness of this resource to the scientific community [42]. The original *T. castaneum* genome assembly was produced by means of capillary DNA sequencing technology, which had limited throughput and high per-base costs [43]. Additionally, to simplify the diploid assembly problem and to meet the high sample input requirements for library preparation, multiple individuals from a highly inbred laboratory strain had to be pooled [42].

Current sequencing technologies have much higher throughput and lower per-base costs, as well as much lower DNA input requirements for library preparation. Some technologies allow for whole-genome sequencing from a single individual, even for insects with very small body sizes [44]. Developments in library preparation and assembly software also allow projects to deal more efficiently with heterozygosity so that the laborious process of creating inbred strains is no longer necessary for genome sequencing. Research efforts have capitalized on these advancements to generate high-quality genomic resources for multiple beetles, including major agricultural pests [45,46,47,48,49,50,51].

Although genomics has the power to characterize the entire gene repertoire of an organism, obtaining a high-quality genome assembly and annotation can be costly and time-intensive. For this reason, many researchers have turned to transcriptomics as an alternative to exploring the genomes of agricultural pests. This approach consists of characterizing the set of genes expressed at a given time using RNA sequencing (RNA-seq). Because only a small portion of the eukaryotic genome is transcribed into messenger RNA (mRNA), transcriptomics requires a much lower sequencing throughput compared to genomics, greatly reducing project costs. This approach has been applied in many agricultural pests for which no genome assembly was available at the time [52,53,54,55].

In the RPW research community, the adoption of omics technologies has been slow, but many advances have already been achieved. In the following sections, we review the existing efforts in this system.

### 3.2. Large-Scale RNA-seq Gene Discovery Studies in the RPW

A number of studies have utilized RNA-seq to identify genes in the RPW, either focusing on the annotation and analysis of a select group of genes or producing a general resource of identified transcripts.

The first study of this type was published in 2013 by Wang et al. [56], and adopted a cDNA sequencing approach. Tissues were obtained from field-collected eggs, larvae, pupae, and adults from Saudi Arabia for RNA extraction and cDNA library construction. The libraries were sequenced on a Roche/454 genome sequencer, and the assembly process yielded 26,765 contigs and 234,901 singletons. Annotation of the resulting contigs was performed in a comparative manner through similarity searches against the NBCI nr database, as well as *T. castaneum* clustered transcripts (Unigenes) and *Onthophagus Taurus* expressed sequence tags (ESTs). These searches identified up to 72%, 50%, and 63% of contigs and 25%, 9%, and 13% of singletons as matching with the nr, *T. castaneum*, and *O. taurus* databases, respectively. The fact that almost 30% of sequences could not be annotated with this strategy might reflect the general lack of sequences from related species in the database at the time.

The next two studies were published in 2015 [57,58]. Yin et al. [57] obtained SOLiD RNA sequencing reads corresponding to five developmental stages of RPW embryogenesis, including four embryonic and one larval stage. The authors mapped their reads to the previously available RPW transcriptome [56], performed gene annotation, and estimated gene expression levels using the RPKM method. This study identified 22,582 genes, 67.3% (15,168) of which were expressed throughout all stages. The number of genes restricted to any developmental stage ranged from 170 to 372. This was the first study to provide a global picture of RPW development, with the identification of developmental stage-associated genes, differentially expressed genes, conserved signaling pathways, and constitutively expressed genes.

In the same year, Yan et al. [58] conducted another transcriptomic study to identify chemoreceptor genes in the RPW that could serve as potential targets for pest mitigation strategies [58]. Chemoreception is a recurrent target of gene identification studies because it is essential for insects to detect host plants, mates, and predators [59]. A cDNA library was prepared from RNA extracted from five female and male RPW adults and sequenced on an Illumina HiSeq instrument, generating about 8 Gb of raw sequencing reads. The resulting assembly and analysis identified 24,439 unigenes from 42,195 assembled contigs. Of these unigenes, 55.19% (13,488) were annotated in at least one public database, and 51.24% (12,523) matched homologous proteins in the nr protein database. In total, 36 putative chemoreceptor genes were obtained and classified within three superfamilies (olfactory, gustatory, and ionotropic receptors). Only three genes were identified as having full-length open reading frames; and homology with *Drosophila grimshawi* and *Bombyx mori* was only identified for two genes. Interestingly, the authors determined the expression profiles of all chemoreceptor genes in multiple tissues through real-time PCR and observed that most were antennae-enriched. However, the number of chemoreceptor genes identified in this study is small compared to other Curculionidae beetles, such as *Dendroctonus ponderosae* [60], raising the possibility that other genes are present that could be identified with additional sequencing.

In 2016, a second attempt to identify RPW chemoreceptor transcripts was performed by Antony et al. [61], in which cDNA libraries were generated from adult antennae and sequenced using the Illumina HiSeq platform. The isolation of RNA from adult antennae is a clever strategy for studies focused on odorant receptors since antennae are the regions where most chemoreceptors concentrate. The authors used a phylogenetic-based approach to annotate 158 genes belonging to 6 chemoreceptor families: 38 odorant binding proteins, 12 chemosensory proteins, 76 odorant receptors, 1 odorant co-receptor, 6 sensory neuron membrane proteins, 15 gustatory receptors, and 10 ionotropic receptors. This study greatly expanded the known odorant receptor repertoire in the RPW.

In 2019, Antony et al. [62] identified cytochrome P450 genes that are known to have major roles in the metabolism of insecticide resistance [62]. In this study, RNA was extracted from the fat body and gut tissues of beetles collected from a date palm tree that received frequent imidacloprid trunk injections. Seventy-seven putative P450 transcripts were identified, including two highly conserved paralogs to *Drosophila Cyp6g1* (*CYP6345J1*) and *Bemisia tabaci CYP4C64* (*CYP4LE1*). RNAi knockdown of select RPW P450 genes resulted in a negative impact on survival in toxicity assays, demonstrating that these genes could be important targets for control strategies. This study was an important addition in that it experimentally validated the involvement of the identified genes in insecticide resistance.

In 2020, a study by Zhang et al. [63] identified 43 putative neuropeptide precursors and 44 G-protein coupled receptors (GPCRs) [63]. The authors utilized two short-read transcriptome data sets, one derived from RPW larvae and the other from pupae. Assembled transcripts consisting of neuropeptides and GPCRs were annotated using BLAST against known genes from *D. melanogaster*, *T. castaneum*, and *Hylobius abietis*. Neuropeptides are involved in many behavioral processes, including reproduction, feeding, learning and memory, and social behavior, and also in many physiological processes, such as development, growth, metabolism, and pheromone biosynthesis. For these reasons, these neuropeptides and their receptors are promising targets for developing new pesticides.

In 2020, two additional transcriptomic studies were conducted with the aim of identifying RPW genes [64,65]. Both studies used the same PacBio long-read isoform sequencing (iso-seq) and Illumina RNA-seq data to generate a full-length transcriptome assembly of the RPW. The input RNA was isolated from larvae, pupae, and both male and female adults. Using the PacBio reads, 63,801 nonredundant transcripts were generated. When mapping Illumina reads to the assembled transcripts, approximately 87%, 89%, 88%, and 88% of the short reads were mapped for male adults, female adults, pupae, and larvae, respectively. Expression analysis found that about 13.45% (8583) of genes are differentially expressed and engaged in metabolic pathways, organ tissue formation, and material transportation in peroxisomes [65]. Approximately 86% of the identified transcripts could be annotated using several public databases [64]; in addition, a total of 9618 long noncoding RNAs, 2084 transcription factors, and 2184 alternative splicing events were identified [64]. The use of long-read sequencing is promising for transcriptome analysis as it is often possible to capture entire transcripts in single reads, reducing the possibility of assembly errors.

In 2021, Engsontia and Satasook [66] annotated gustatory receptors in publicly available RPW genomic and transcriptomic data and were able to identify 65 gustatory receptors, including 49 bitter, 9 sugar, and 7 carbon dioxide receptors [66]. The authors found a large amount of seemingly recent gene duplications in the gustatory receptor family, which should be taken with caution since the reference genome used in their analysis was found to include haplotype duplication artifacts (see Section 3.3).

### 3.3. Whole Genome Sequencing in the RPW

Sequencing and generating draft assemblies for non-model species, such as the RPW, has been hindered by the abundant heterozygosity present in diploid sexual populations. However, the rapid development of new approaches, such as linked-read and long-read sequencing, along with appropriate assembly tools, has allowed the generation of de novo genomes for diploid systems with high quality and contiguity, and thus more accurate representations of the genome structure and gene content [67]. There are currently three assembly drafts and one functional annotation publicly available for the RPW.

The first publicly available RPW draft genome sequence was published in May 2020 [68]. DNA was extracted from a pool of male and female adult RPWs collected from an infested field. A total of 190 Gb of raw sequencing reads were generated using 10× Genomics linked-read and standard Illumina paired-end sequencing technologies. The assembly process involved producing several intermediate assemblies that were merged and finally scaffolded using the *T. castaneum* genome as a reference. This hybrid assembly (NCBI accession number GCA_012979105.1) is 782 Mb long and has a contig N50 of 23.8 Kb and a scaffold N50 of over 64 Mb. The high scaffold N50 metric could be artifactual since taking *T. castaneum* as the scaffolding reference assumes the colinearity of the two genomes, although the species diverged over 160 million years ago [69]. In addition, the authors report somewhat intriguing BUSCO scores, with ∼50% of BUSCOs duplicated, 12% fragmented, and 15% missing [68]. A high proportion of BUSCO core genes are expected to be present, complete, and single-copy in any genome, and deviations from this expectation are indicative of issues with assembly quality. In fact, Dias et al. [70] have shown that this hybrid assembly [68] includes a high degree of artifactually duplicated sequences resulting from uncollapsed haplotypes, likely stemming from the use of a heterozygous sample containing multiple individuals. This implies that the unusually high rate of gene family expansion reported by Hazzouri et al. [68] should be taken with caution and/or reassessed.

In September 2021, a second draft genome was published for the RPW [70]. This assembly was produced from a single RPW larva using 10× Genomics linked reads. The 10× linked-read technology is able to resolve divergent haplotype regions from a diploid sample, separating the assembly into two pseudo-haplotypes. This haplotype-resolved assembly (GCA_014462685.1) is 589.40 Mb long with a contig N50 of 37.9 Kb and scaffold N50 of 472 Kb. Analysis of single-copy orthologs revealed high completeness, with 98.1% complete and 96.2% complete and single-copy BUSCOs being present. Prediction of protein-coding genes was conducted using BRAKER [71], utilizing predicted proteins from several insects as well as multiple publicly available RPW RNA-seq reads as inputs. Annotation of the assembly predicted 25,382 transcripts that clustered into 23,413 genes. Over 88% of exon-intron junctions were validated by spliced alignments, indicative of accurate annotations. About 86.7% of the predicted proteins could be annotated in existing databases. This assembly was finally used to cross-validate previous resources, such as the chemosensory gene annotation from Antony et al. [61], cytochrome P450 genes from Antony et al. [62], neuropeptides and GPCRs from Zhang et al. [63], and iso-seq data from Yang et al. [64]. These analyses validated previous gene assembly/annotation but also revealed potential shortcomings of previous RNA-seq-based approaches, such as transcripts assembled on the incorrect strand and the presence of putative artifactual transcripts that were not supported by assembly or short-read mapping.

An additional draft genome of the RPW was reported by Manee et al. [72], for which data were generated using Illumina paired-end sequencing. The DNA for this assembly were obtained from a single female adult RPW. The total length of the assembly was approximately 1121 Mb, and it had a modest N50 of nearly 8 kb. This draft genome was used for the analysis of simple sequence repeats and was made publicly available through zenodo.org. Available at https://doi.org/10.5281/zenodo.6878576 (Accessed 31 January 2023).

Interestingly, the sizes of available RPW genome assemblies vary greatly, from 589.40 to 1121.36 Mb [68,70,72]. Although this observation could indicate intraspecific variation in genome size [73], other explanations have been proposed. Hazzouri et al. [68] report flow cytometry estimates of genome size in the RPW to center around 700 Mb [68]. Dias et al. [70] have argued that based on these flow cytometry estimates, one would expect an assembly size shorter than 700 Mb since highly repetitive heterochromatic regions are often not assembled completely [70].

### 3.4. Re-Analysis of Available Transcriptomic and Genomic Resources Using BUSCO

Since comprehensive benchmark software was not available at the time when the first RPW transcriptomic studies were published, we have re-analyzed available transcriptomic/genomic data for the RPW using BUSCO, aiming at providing an updated picture of the available data. We utilized the public server gVolante [74] BUSCO v5 and the arthropod gene set from OrthoDB v10 [75,76]. Unfortunately, many datasets generated previously have not been deposited to a public database and thus can not be re-analyzed.

According to our analysis, all available RPW resources have reasonably high BUSCO completeness, with >80% completeness for all datasets (Table 1). The amount of duplicated BUSCOs is generally low, with the exception of the Hazzouri et al. [68] genome assembly (discussed previously) and the Yang et al. [64] long-read transcriptome data [64]. BUSCO duplication in transcriptome assemblies is not generally of concern as it might simply reflect the presence of multiple isoforms of the same gene.

The amount of fragmented and missing BUSCOs is highest in the transcriptome assembly published by Wang et al. [56], with 10.6% fragmented and 8.1% missing genes (Table 1). This result is somewhat expected as this was the first transcriptome sequencing performed for the RPW in 2013, and the sequencing platform used (Roche/454) had lower throughput/quality than newer Illumina instruments [77].

### 3.5. Metagenomics of Gut Microbiota in the RPW

Insect gut communities are dynamic and can be determined by factors such as developmental stage, host phylogeny, and environmental habitat [78,79,80]. Moreover, an organism’s microbiota influences aspects of its fitness, such as growth, survival, development, and adaptation [81]. DNA sequencing enables rapid characterization of the microbiome, which can reveal the potential functions of microorganisms within their communities and their potential services to the host insect, such as roles in insecticide resistance. As the RPW spends the entire larval stage of its life cycle inside the trunk of the palm tree, the intestinal microbes of the weevil larva are essential in enabling its feeding on the fibrous tissues of the trunk [82]; thus, understanding the RPW microbiota at the molecular level is crucial to understanding weevil biology and for the prospection of biocontrol strategies.

Few metagenomic studies have been published on the gut microbial community of the RPW [80,82], although other studies have adopted an amplicon sequencing approach to estimate microbial diversity in the gut [78,83]. Jia et al. [82] used pyrosequencing to characterize the taxonomic profiles of the RPW gut microbiota at different time points [82]. Specifically, total DNA was extracted from the gut contents of larvae samples collected at diverse seasonal times (March, July, and November) and from adult samples collected in March and May, which were later combined into one sample named “adult”. The Roche/454 GS FLX instrument was used for shotgun and amplicon sequencing and generated 5.3 million raw reads (approximately 2 GB). *Lactococcus lactis* was found to be the most frequent gut microbe in July larvae and in adult samples, and *Klebsiella pneumoniae* in March and November larvae. Twenty-three percent of the species in November’s microbial population have been associated with cellulolytic digestion; the dominant bacteria during that period belonged to the *Proteobacteria* phylum, such as *Enterobacter*, *Klebsiella*, and *Pantoea*, which is recognized to have cellulolytic capabilities. In addition, the microbial groups identified in November and July were found to have roles in the symbiotic relationship between host and microbiota. The effect of high-temperature stress on microbial communities was also investigated.

In 2017, Muhammad et al. [80] investigated the bacterial entomotype and dynamic alterations in the RPW gut, as well as any potential physiological functions [80]. DNA was isolated from ten gut extracts representing RPW larvae, pupae, and adults collected from coconut trees, and 16S rRNA metagenomic sequencing was carried out on the Illumina MiSeq platform. The predominant phyla were *Proteobacteria* and *Firmicutes*, consistent with previous reports [78,82]. At the family level, the most abundant families were the *Enterobacteriaceae*, *Streptococcaceae*, *Lactobacillaceae*, *Enterocococcaceae*, *Enterobacteriaceae*, and *Entomoplasmataceae* for all RPW life stages, an observation similar to the existing literature [82,83]. Seven bacterial species from the genera *Klebsiella*, *Serratia*, *Enterobacter*, and *Citrobacter* were demonstrated to break down cellulose in the gut of RPW larvae. Taken together, this and other studies suggest a high degree of stability for microbial communities in the RPW gut, with only slight variations in abundance across several life stages and host plants [80,82,83].

### 3.6. Repetitive DNA in the RPW Genome

The eukaryotic genome includes a significant portion of repeats. These can include dispersed repeats, mainly composed of transposable elements and segmental duplications, and tandem repeats such as satellite DNAs (satDNAs), minisatellites, and microsatellites [84]. Aside from their eventual exaptation and potential functions [85], DNA repeats are important structural components of eukaryotic genomes, and understanding their organization and evolutionary dynamics will prove paramount to illuminating the history of eukaryotic genome architecture as a whole [86].

The study of repeats in the RPW genome is in its infancy, but the use of genomics has greatly accelerated its progress. In the past year, two studies were published using short-read sequencing to characterize microsatellites and satDNAs in the RPW [72,87]. Manee et al. [72] generated a new low-coverage draft genome of the adult female RPW to search for microsatellite content (1–6 bp long) and compare it to other beetles from the Curculionidae family, including the two publicly available draft genomes of the RPW [72]. Consistent search parameters were used to analyze each draft genome. The results showed that the identified SSR content conforms to the degree of coverage, with values of 0.13–0.36% for the RPW draft genomes. There was a slight variance in the SSR proportions identified for other weevil species, ranging from 0.02% to 1.44%. In addition, the number of SSRs and genome size was found to be significantly positively correlated, which is consistent with prior results for 136 insects [88]. Finally, the most frequent SSR types were mono- to trinucleotide SSRs, and tetra- to hexanucleotide SSRs were the least abundant.

Montiel et al. [87] carried out the first attempt to characterize satDNAs of the RPW genome [87]. These authors found 121 satDNA families in the RPW, comprising 25% of its genome, the highest diversity of satDNA families of any insect to date. Interestingly, Montiel et al. [87] found that the most abundant satDNAs can be detected as dispersed signals in euchromatin, in addition to the more classic heterochromatic locations. The authors also analyzed the transcription of satDNAs across RPW life stages and found stage-specifically transcribed families, suggesting a potential role for these transcripts in developmental processes [87].

Hazzouri et al. [68] used RepeatModeler and found that 45% of the 782 Mb assembled genome consisted of transposable elements, primarily mariner-like elements [68].

### 3.7. Mitochondrial Genome of the RPW

The mitochondrial genome is an excellent and informative model for molecular evolution, comparative genomic research, and genetic and phylogenetic analysis because of its maternal inheritance mode, high evolutionary rate, lack of introns, and limited recombination [89,90]. A typical metazoan mitochondrial genome is a circular molecule that encodes 37 conserved genes, including 13 protein-coding genes, 22 transfer RNA genes, 2 ribosomal RNA genes, and a control region [89]. As far as we know, Zhang et al. [91] was the first study to sequence, assemble, and annotate the complete mitochondrial genome sequence of the RPW [91]; this sequence was deposited to GenBank under accession number KT428893. The mitochondrial DNA was extracted from adult RPWs collected from trees in Lishui city, Zhejiang province, China. As this study is quite old, the sequencing instrument used was the 454 sequencing system, and the assembly method used was MIRA4 [92]. The assembled mitogenome was 15,924 bp in length with 25.6% GC content and contained 13 protein-coding genes (PCGs), 21 transfer RNAs, 2 ribosomal RNAs, and a control region (D-loop region) [91]. A second mitochondrial genome of the RPW (GenBank accession number CM025099) was recently deposited, with a length of 17,157 bp. These data were generated by the same group and are part of their publication in 2021 [70].

## 4. Opportunities and Challenges of Practical Uses of RPW Omics Research for Management Approaches

Due to the shortcomings of traditional RPW management techniques over the last 30 years, many omics studies have been carried out on the RPW with the hope of improving technologies for managing this pest in the field. However, truly understanding the RPW pest-plant dyad will also require understanding the palm trees it attacks. For instance, proteomics of palms attacked by the RPW may help identify opportunities for control and early detection [93]. Additionally, the identification of binding sites/receptor proteins used for pheromone/gustatory reception could be used to fabricate sensors that could detect weevils inside date palm trunks [61,70].

Genomic and transcriptomic data already available for the RPW may also be useful in identifying genes suitable for targeting with genetic/genomic engineering tools, such as RNAi and CRISPR/Cas-based approaches [93]. RNA interference has been successfully used to control the western corn rootworm *Diabrotica virgifera virgifera* [94]. This technology is suitable for *R. ferrugineus*, where both adults and larvae could be targeted using dsRNAs [93]. The CRISPR-based precision-guided sterile insect technique (pgSIT) is also promising for the management of the RPW. This technique was first developed by Kandul et al. [95] in the fruit fly *Drosophila* and employs a dominant genetic process that imposes genes essential for female viability and male sterility simultaneously, resulting in seamless sexing and sterilization. Eggs produced with this technique are released into the field, and upon hatching, only sterile males emerge [95].

Laboratory experiments have successfully used RNAi to silence several genes in the RPW, namely α-amylase, V-ATPase, catalase, and ecdysone receptors [96,97]. The silencing dsRNA was administered to RPW larvae through topical application, microinjection, and via artificial diet, with all three methods of application inhibiting larval growth and inducing mortality. All RNAi/CRISPR techniques could also benefit from improved reference genomes and some knowledge of the genetics of invasive RPW populations. To this day, population genetics of the RPW are restricted to the use of a single/few markers, mostly mitochondrial genes and microsatellite loci [98,99,100,101]. In this context, whole-genome sequencing of multiple field-collected individuals from diverse locations would be an important step toward understanding RPW population history and genetic diversity.

Additional approaches to using omics to manage RPW include the use of transgenic insect viruses, namely Baculoviruses [102], or microbes (symbionts) that are maternally inherited. A prominent symbiont in this respect is Wolbachia, which mediates infertility in insects [103]. High-quality DNA sequencing of diverse isolates of wild RPW could also aid in the identification of naturally occurring symbionts that could be exploited for pest control.

Host-plant resistance against the RPW is not very well understood and offers a whole new area of exploration wherein traditional plant breeding techniques coupled with advanced molecular-based breeding techniques could be used to induce resistance against the RPW in popular palm cultivars. The entire genome of the date palm cultivar ‘Khalas’ has been sequenced [104,105], which could facilitate the integration of genetic engineering techniques into breeding programs, overcome the current constraints to conventional breeding of date palms, and help to incorporate desirable traits of yield, quality, and resistance to abiotic and biotic stresses [106].

Some challenges that still face the application of omics-based approaches for controlling the RPW include the identification of suitable gene(s) for RNA-guided dominance and developing a robust CRISPR/Cas system for *R. ferrugineus*. Alongside these, the technical difficulties in the production of transgenic date palms coupled with the lower tendency of consumers to accept such genetically modified crops [93] represent other great challenges that may hinder the adoption and application of RPW-omics in management strategies.

## 5. Conclusions

Despite the success of omics approaches in advancing pest biological research, research communities have been slow to adopt these techniques, and pest management programs have traditionally focused on applied rather than basic research. Nonetheless, unexpected discoveries and conceptual advancements in our understanding of pest biology are being made due to the enhanced knowledge and insights garnered through pest omics. Omics data are also expected to boost our understanding of RPW evolution and thus to open comparative genomics avenues of research. For example, knowing more about the geographical distribution of RPW genetic variation will help reveal patterns of dispersal among palm tree farms that can inform transportation and treatment guidelines.

As pest research moves toward a systems biology paradigm, the integration of an ever-growing number of data sources will help in future-proofing pest control and protecting our food supply and biodiversity.

## Figures and Tables

**Table 1:** Single-copy ortholog statistics for available transcriptome and genome assemblies of the red palm weevil.

Resources	BUSCO				
	Complete	Single-Copy	Duplicated	Fragmented	Missing
Transcriptomes					
Wang et al. [56]	81.3	71.1	9.6	10.6	8.1
Antony et al. [61]	95.9	94.8	1.1	2.7	1.4
Yang et al. [64]	92.8	39.5	53.3	1.5	5.7
**Genomes**					
Hazzouri et al. [68]	93.3	55.6	37.7	2.2	4.5
Dias et al. [70]	98.6	96.5	2.1	1.1	0.3
Manee et al. [72]	94.6	92.2	1.7	4.1	1.3

## Data Availability

All data produced in this study are available in the manuscript body.
